# Audit of the Handover in Resident Doctors’ First On-call Rota

**DOI:** 10.1192/bjo.2025.10607

**Published:** 2025-06-20

**Authors:** Ayah Ibrahim

**Affiliations:** Lancashire and South Cumbria NHS Trust, Chorley, United Kingdom

## Abstract

**Aims:** Handover is ‘the accurate reliable communication of task-relevant information across shift changes or between teams thereby ensuring continuity of safe and effective working’. Handover is of critical importance; this ensures high quality care of our patients. Therefore this audit **Aims:**

To explore the compliance rates for completing handover in the junior doctor on-call rota in Blackburn Area.

To explore the current practice of whether handover is completed in person, whether it is documented or whether these are done together.

To use this audit to implement changes to improve the current handover practice.

**Methods:** A form was printed and put in the on-call room, doctors coming into handover were requested to tick if they received handover face to face, written, both, or none, on that particular shift.

If a doctor forgot to fill the form, they were followed up by email.

The data was collected over a three-week period covering 57 shifts changeover.

Data analysis was qualitative and quantitative.

**Results:** 57 shifts changeover, over 21 days.

(14%) 8 shifts changeover with no handover done at all.

(28%) 16 shifts changeover only with complete handover (in person and written).

(23%) 13 shifts changeover with only in person handover with no documentation.

(35%) 20 shifts changeover with only written handover with no in person handover completed.

**Conclusion:** This audit was able to look into the purpose it was designed for. It identified an area of improvements to work on, to achieve good practice and to maintain patient’s safety. One of the findings of this audit, is that on some occasions, doctors have indicated they have received a written handover, however it was not found in the assigned handover teams channel, therefore, it is possible that the handover was sent in another way, i.e. in private chat in teams or emails etc. This audit has concluded the importance of keeping handover in one assigned place, so records can be easily accessible to other team members when needed.

Recommendations were made by meeting the locality college tutor, discussion was carried around notifying all doctors in training about the importance of handover in induction meetings and reminder emails. A poster was also designed and put in doctors’ on-call room.

This audit is to be repeated for monitoring purposes. with a recommendation of having senior doctors involvement in monitoring.

There have been some limitations in doing this audit, for instance, the handover for twilight shifts could have been only partial when completed and this audit did not go into details whether full handover was received from all wards or not. This audit also did not look into the quality of the handover itself – whether details are clear and information needed is provided.

